# The Prevalence of Dry Eye Syndrome’s and the Likelihood to Develop Sjögren’s Syndrome in Taiwan: A Population-Based Study

**DOI:** 10.3390/ijerph120707647

**Published:** 2015-07-08

**Authors:** Ju-Chuan Yen, Chia-An Hsu, Yu-Chuan (Jack) Li, Min-Huei Hsu

**Affiliations:** 1 Graduate Institute of Biomedical Informatics, College of Medical Science and Technology, Taipei Medical University, Taipei 110 Taiwan; E-Mail: m701061@gmail.com; 2 Department of Ophthalmology, Taipei City Hospital, Zhongxiao Branch, Taipei 115, Taiwan; 3 School of Medicine, National Yang-Ming University, Taipei 112, Taiwan; E-Mail: johnhsu129@gmail.com; 4 Ministry of Health and Welfare, Taipei 115, Taiwan

**Keywords:** prevalence, dry eye syndrome (DES), Sjögren’s syndrome (SS), National Health Insurance Research Database (NHIRD)

## Abstract

*Background*: Dry eye syndrome (DES) is one of the key clinical features and possibly an early clinical presentation of Sjögren’s syndrome (SS). We explore DES prevalence and assess the likelihood of DES patients to develop SS in Taiwan through the National Health Insurance Research Database (NHIRD). *Methods*: Through a cohort comparison study, longitudinal data from the NHIRD (2000 to 2008) in Taiwan was used to probe the prevalence of DES and the odds that DES patients would later develop SS. *Results*: The prevalence of DES in the present study is 4.87%. The incidence rates of developing SS were 4.8% for the DES group and 1.5% for comparison group. The median age and interquartile range of DES and comparison patients was 49.8 (10) and 48.7 (15) years old, respectively. The crude hazard ratio (with 95% confidence interval) for DES patients to develop SS was 3.13 (3.10–3.50) for the DES group, and the adjusted hazard ratio (with 95% confidence interval) was 3.64 (3.43–3.87). The observation period and interquartile range for DES and comparison patients to develop SS later were 1418 (781–2316) *versus* 1641 (971–2512) days respectively. *Conclusions*: DES patients carried a higher risk for developing SS (hazard ratio 3.13) and presented for SS 3.88 years earlier than comparison group patients in this study.

## 1. Introduction

Dry eye syndrome (DES) is one of the most common disorders in ophthalmology services. Surveys show different figures regarding prevalence, from around 5% to 35% [1,2], differences that might be due to sampling methods and population demographics. It was believed that prevalence of dry eye syndrome is higher in Asian populations than those in Western countries, based on ethnic differences [3]. Dry eye syndrome is significant, not only because DES typically makes patients suffer from dry sensation, redness, burning, photophobia, and even fluctuating blurry vision, which decrease patients’ life quality, but also because it could be a presentation of autoimmune diseases, such as Sjögren’s syndrome (SS) or other autoimmune disorders.

Dr. Henrik Sjögren [4,5] first published in 1933 a case series observation of 19 females with dry eye symptoms; most of them had rheumatoid arthritis. After that, other clinical features were unveiled accordingly, including dry mouth and dry mucosal membranes. Many studies have echoed the female preponderance of Sjögren’s syndrome and associations with autoimmune disorders. Sjögren’s syndrome could be primary or secondary to other autoimmune disorders such as systemic lupus erythematous, dermatomyositis, systemic sclerosis, and so on. The diagnostic criteria were based on American-European Census Group (AECG) in 2002, mainly through clinical presentations and laboratory results. Besides, there is evidence that Sjögren’s syndrome would increase the risk of developing malignancy especially non-Hodgkin’s lymphoma B cell type [6]. Although many studies have been published on the association of dry eye syndrome and Sjögren’s syndrome, a population study among them is rare. Therefore, we proposed to examine the prevalence of dry eye syndrome and the association of the DES and SS through the National Health Insurance Research Database in Taiwan.

In Taiwan, the government launched the national health insurance program as a mandate on March 1st 1995. Today, the coverage rate is around 99%, which means almost all of the population is covered by national health insurance [7]. A nationwide population study using a longitudinal case-controlled cohort study was conducted to examine whether dry eye syndrome was a risk factor for Sjögren’s syndrome and to compare the hazard ratio between the cohorts. The Longitudinal Health Insurance Database 2000 (LHID2000) is a sub-dataset of the National Health Insurance Research Database (NHIRD), which includes all claims data (from 1996 to 2008) of one million beneficiaries who were randomly selected from the system in 2000. There was no significant difference in age, sex, or average insured payroll-related premiums between the sample group participants and insured individuals.

## 2. Materials and Methods

### 2.1. Selection of Patients and Variables

This study consisted of all patients (n = 48,704) in the Longitudinal Health Insurance Database 2000 who had been diagnosed with dry eye syndrome (DES, ICD-9-CM codes 375.15) [8,9] and seen in ambulatory (including emergency) and inpatient care from 1 January 2000 through 31 December 2008. The sample size of comparison group (*i.e*., patients not diagnosed with DES) was set at three times the DES patients group. Comparison patients were randomly selected from the dataset. The patients included in the study and the comparison group patients were matched by sex, age, and the index date of the ambulatory care visit (including outpatient clinic and emergency department) or date of hospitalization for the initial diagnosis of DES patients. Among these datasets, we further coded all patients who had ever developed Sjögren’s syndrome (ICD-9-CM codes 710.2) after a diagnosis of dry eye syndrome. We also recorded demographic data, such as, sex and age. This study was approved by the institutional review board of Taipei Medical University, Taiwan. Since this study analyzed deidentified data, the review board waived the requirement for written informed consent from the patients involved.

### 2.2. Statistical Analysis

SAS for Windows 9.3 (SAS Institute, Inc., Cary, NC, U.S.A.) was used for this study. Descriptive statistical analyses were done to compare the characteristics of the two cohorts in terms of demographic characteristics and the risk of developing Sjögren’s syndrome. We compared DES patients with the comparison group concerning the risk of developing Sjögren’s syndrome by estimating the crude hazard ratio with conditional logistic regression. Kaplan-Meier analysis was used to calculate the cumulative incidence rates of developing Sjögren’s syndrome in each of the cohorts and the log-rank test was used to analyse the differences between the survival curves. Thereafter, we performed separate Cox proportional hazard regressions to compute the Sjögren’s syndrome-free survival rate after adjusting for possible confounding factors such as age and sex. Statistical significance was set at *p* ≤ 0.05.

## 3. Results

### Demographic Data

Between 2000 and 2008, 48,704 dry eye syndrome patients and 128,542 comparison patients, with age- and sex-matched controls were recruited after excluding ineligible subjects. The ratio did not arrive at 1:3 as the DES case number is pretty large in this one million beneficiaries database, and the database cannot provide enough cases for 1:3 ratio, and only arrived at 1:2.64. The median age and the interquartile range of the DES patients was 49.8 (10) years old, respectively and for the comparison group it was 48.7 (15) years old, respectively. The sex ratios of the two groups were as follows: males, 29.9% and females, 70.1% in the DES group; in the comparison group—males, 33.9% and 66.1% females (see Table 1). 

For the dry eye syndrome group (n = 48,704), 2329 patients (4.8%) later developed Sjögren’s syndrome; in the comparison group (n = 128,542) 1927 patients (1.5%) later developed Sjögren’s syndrome. Thus, of 177,246 cases, 4256 patients (2.4%) developed SS. The difference between the dry eye syndrome groups concerning their risk of developing SS was statistically significant (*p* < 0.0001). The median observation period and interquartile range between the two groups to develop the SS was 1418 (781–2316) days in the patient group, and 1641 (971–2512) days in the comparison group. The observation period for later developing SS differed in length when comparing the DES group to the comparison group. This was statistically significant: the *p* value was <0.0001 (see Table 2).

**Table 1:** Comparison of dry eye syndrome (DES) and comparison groups concerning later development of Sjögren’s syndrome (SS) with respect to characteristic demographics.

Variable	DES Patients (*n* = 48,704)		Comparison Group (*n* = 128,542)		*p* Value
	n	%	n	%	
Age, median ( IQR ^a^)	49.8 (10)		48.7 (15)		
Gender					<0.01 *
Male	14,543	29.9	43,624	33.9	
Female	34,161	70.1	84,918	66.1	
SS	2329	4.8	1927	1.5	<0.0001 **
Observation duration (days, median, IQR ^a^)	1418	781–2316	1641	971–2512	<0.001 **

^a^ IQR: Interquartile range; * Indicates *p* < 0.01; ** Indicates *p* < 0.001.

**Table 2:** Crude and adjusted hazard ratios for developing Sjögren’s syndrome (SS) among patients with dry eye syndrome (DES) and comparison group during 2000–2008 follow-up.

Development of SS	Total		DES		Comparison Group	
	No.	%	No.	%	No.	%
Yes	4256	2.4	2329	4.8	1927	1.5
No	172,990	97.6	46,375	95.2	126,615	98.5
Crude HR (95% CI)	–		3.30 (3.10–3.50)		1.00	
Adjusted ^a^ HR (95% CI)	–		3.64 (3.43–3.87)		1.00	

^a^ Adjustments were made for sex and age.

**Figure 1:**
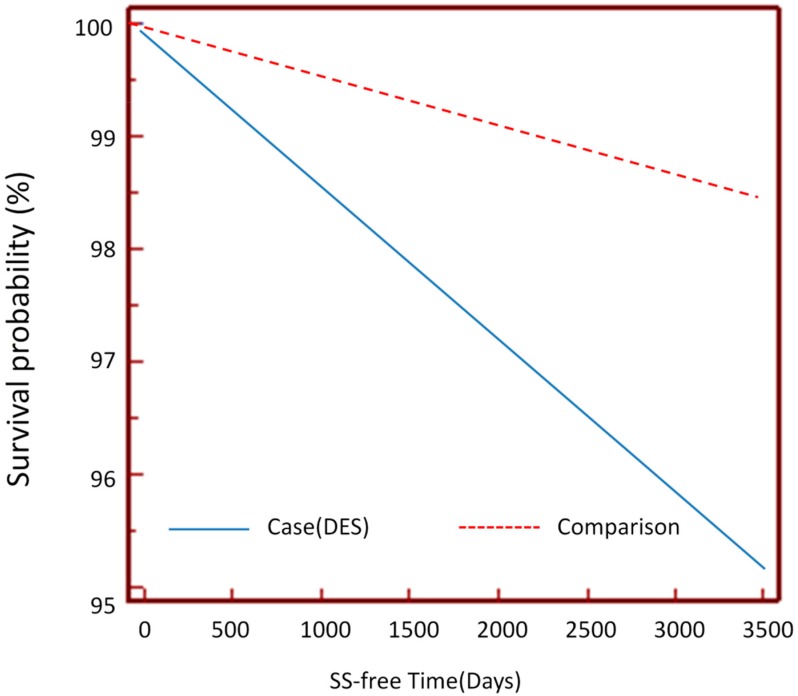
Kaplan-Meier Survival analysis of DES patients against SS-free time.

Among the cohorts, the crude hazard ratio using logistic regression with a 95% confidence interval for the DES patients to develop SS is 3.30 (3.10–3.50); the adjusted hazard ratio by Cox proportional regression model is 3.64 (3.43–3.87). The adjusted factors included age and sex. Kaplan-Meier survival analysis was conducted to examine the cumulative incidence rates of the two cohorts developing Sjögren’s syndrome, and a log rank test was used to probe the differences between the survival curves. The results (see Figure 1) revealed statistically significant differences between the DES and comparison group, *i.e*., *p* < 0.0001. We also compared the ratios of females to males in the DES and comparison groups. The sex difference between DES patients was very obvious at 2.34 (70.1:29.9), which meant female DES patients outnumbered their male counterparts by 2.34 times.

## 4. Discussion

The prevalence of dry eye syndrome in Taiwan in this study was 4.87%, the median age and interquartile range was 48.9 (10) years old, with female preponderance and the ratio of female to male was 2.34 (70.1:29.9). In the United States’ survey among women and men [10,11], the prevalence was 7.28% for women and 4.37% for men, the prevalence in female was obviously higher than that of their male counterparts, but the research subject inclusion criterion was aged ≥50 years old. Our study comprises a whole population with full range of ages and, possibly, that is why the prevalence rate for the Taiwan population is lower. In other studies, like the Japanese study in Kuomi [3], the prevalence of clinical diagnosis of dry eye syndrome among men and women was 12.5% and 21.6%, respectively; the female prevalence was still higher. This study also noted female dominance in cases of dry eye syndrome. Another prevalence study of dry eye syndrome in Indonesia [12] showed 27.5% among adults. Another population-based study in Taiwan [13] reported the prevalence dry eye syndrome in the Chinese elderly’s was 33.7%, yet the inclusion criterion was aged ≥ 65 years old and the location was in Shih Pai, which is a small area over northern Taiwan; however, that was relatively a smaller scale study and with the older age population as compared to ours. To sum up, the present nationwide population study in Taiwan, which could be representative of any Asian country, revealed the median age was 48.9 years old and with female preponderance. It echoes previous studies that have found the fourth or fifth decade of life could be the peak age of dry eye syndrome. The sex ratio difference showing female dominance is believed to be due to estrogen overproduction or androgen underproduction. 

Sjögren’s syndrome is an autoimmune disorder that involves multiple organs. The key feature of Sjögren’s syndrome is the lymphocyte infiltration of exocrine glands and other organs. Among them, lacrimal gland and salivary gland involvement by lymphocytes lead to dry eye and dry mouth and are crucial presentations of SS, and so the eye might be the first organ to be affected by and thus serve as a diagnostic feature of Sjögren’s syndrome. On the other hand, a higher prevalence of hyperlipidemia, cardiac arrhythmias, migraines, pulmonary circulation disorders, and hepatitis B was observed in patients with SS and DES patients [14,15].

In clinical settings, it’s not common for ophthalmologists to screen for Sjögren’s syndrome, because extra-glandular ocular involvement is not very frequent and ophthalmologists are not familiar with systemic extra-glandular involvement, such as occurs in pancreatitis and hepatitis and even malignancies. 

Akpek *et al*. examined a longitudinal cohort of 163 patients with Sjögren’s syndrome from tertiary-referred hospitals in the United States. The study revealed that 98% of the patients had a history of dry eye for an average of 10.4 years (median, 7.9 years) before the presentation of Sjögren’s syndrome [16]. In our present study, the average time of DES to develop into SS is 3.88 years, many fewer years as compared to the Akpek study. Up to 55% of patients with a vision-threatening ocular finding did not have a diagnosis of SS at presentation. Therefore, the authors also advocated that the ophthalmologists should be aware of the disease association of dry eye syndrome with full development of Sjögren’s syndrome. This would help contain possible complications and disease progression both in the ocular entities like sterile corneal necrosis [17,18], uveitis [19,20], and scleritis [21,22], as well as extra-glandular involvement, such as from pancreatitis, thyroid gland involvement or non-Hodgkin’s lymphoma [6], especially B cell type in the systemic fields simultaneously. It was believed that around one-tenth of dry eye syndrome patients who presented with clinically obvious symptoms and signs were having SS. Of those patients, only one third received a diagnosis of SS at DES clinical presentation [18]. Therefore, we echo the authors’ perspective that Sjögren’s syndrome is underdiagnosed as the presentations were diverse and not at full scope in the beginning, and so underdiagnoses and delay of treatment of Sjögren’s syndrome were not rare and might cause further disease burden to the patients and society. 

This study was conducted based on a single-payer, longitudinal, national health insurance database in Taiwan to probe the hazard ratios of DES as risk factors for SS development. Were it not for this NHIRD longitudinal dataset, it would be very difficult to collect enough cases to conduct the research because of time-consumption and cost issues. And since it is a national health insurance database, it is a population-based study, so the results are more robust and convincing without selection bias.

On the other hand, the claims data of the database could not show important clinical features such as the Schirmer’s test, tear film breakup time, and corneal vital stain results. It could not show personal health records, such as, salivary and lacrimal gland biopsy reports, or indicators such as body mass index, individual diet and exercise routines, or important life events, which are important in interpreting the etiology of SS. Our study did not adjust comorbidities such as hyperlipidemia, cardiac arrhythmias, migraines, pulmonary circulation disorders, and hepatitis B as the confounding factors, and the confounding effects could influence the power of interpretation in present study. To use ICD-9-CM codes 375.15 to recruit our study group alone and not to add other disease code such as 370.33 of keratoconjunctivitis sicca was becasue patients with diagnoses of keratoconjuctivitis sicca mostly have known diagnosis of SS, but this coding could decrease our study group population number and influence the power of interpretation in our study. Finally, to use ICD-9-CM codes 375.15 for DES and ICD-9-CM codes 710.2 for SS also has the limitations for the verification towards disease diagnoses as these disease codes were put by practicing physicians for claims data. And these are limitations of present study.

## 5. Conclusions 

Taiwan’s national health insurance database was used to examine the association of dry eye syndrome to the later development of Sjögren’s Syndrome. The results revealed DES is associated with higher risk of later developing SS, as the crude HRs with 95% confidence interval was 3.30 (3.10–3.50), and the adjusted HRs with 95% confidence interval was 3.64 (3.43–3.87). Dry eye syndrome occurred before Sjögren’s syndrome in some cases, presenting around four years earlier in clinical presentation—the SS-survival free day and interquartile range for DES was 1418 (781–2316) days. We suggest ophthalmologists detect underlying causes of DES to avert Sjögren’s syndrome to increase rates of early diagnosis of SS and to contain disease disabilities, which include extra-glandular involvement ocularly and systemically.
